# Nuclear localisation of nuclear factor-kappaB transcription factors in prostate cancer: an immunohistochemical study

**DOI:** 10.1038/sj.bjc.6602796

**Published:** 2005-10-04

**Authors:** L Lessard, L R Bégin, M E Gleave, A-M Mes-Masson, F Saad

**Affiliations:** 1 Centre de recherche du CHUM, and Institut du cancer de Montréal, 1560 Sherbrooke Est, Montréal, Québec, Canada H2L 4M1; 2 Service d'anatomopathologie, Hôpital du Sacré-Cœur de Montréal, 5400 boul. Gouin Ouest, Montréal, Québec, Canada H4J 1C5; 3 The Prostate Centre, Vancouver General Hospital, University of British Columbia, D-9, 2733 Heather Street, Vancouver, British Columbia, Canada V5Z 3J5; 4 Département de médecine, Université de Montréal, Québec, Canada; 5 Département de chirurgie (urologie), CHUM-Notre-Dame, 1560 Sherbrooke Est, Montréal, Québec, Canada H2L 4M1

**Keywords:** prostate cancer, NF-*κ*B, immunohistochemistry, tissue microarray, Gleason grade

## Abstract

Several reports suggest that the canonical nuclear factor-kappaB (NF-*κ*B) pathway is constitutively activated in a subset of prostate cancer cells. However, except for RelA (*p65*), little is known about the status of NF-*κ*B transcription factors in prostate cancer tissues. To clarify the status of NF-*κ*B subunits, we analysed the expression and subcellular localisation of RelA, RelB, c-Rel, p50, and p52 on tissue array sections containing respectively 344, 346, 369, 343, and 344 cores from 75 patients. The subcellular localisation of NF-*κ*B factors was tested against relevant clinical parameters (preoperative prostate-specific antigen, pathological stage, and postoperative Gleason grade). With the exception of c-Rel, each subunit was detected in the nucleus of cancer cells: significant nuclear expression of RelB, RelA, p52, and p50 was seen in 26.6, 15.6, 10.7, and 10.5% of cores, respectively. Surprisingly, cores expressing both nuclear RelA and p50 canonical pathway proteins were less frequently observed than cores expressing other subunit combinations such as RelB–p52 and RelA–RelB. In addition, the nuclear localisation of RelB correlated with patient's Gleason scores (Spearman correlation: 0.167; *P*=0.018). The nuclear localisation of both canonical and noncanonical NF-*κ*B subunits in prostate cancer cells suggests for the first time that different NF-*κ*B pathways and dimers may be activated in the progression of the disease.

In Caucasian men, prostate cancer is the most frequently diagnosed cancer and a leading cause of cancer death. Presently, the majority of clinically localised tumours are detected early and treated aggressively even though some early-stage tumours can remain latent and may not require aggressive therapy. New prognostic tools are needed to distinguish between low- and high-risk tumours in order to tailor treatment strategies. Understanding the molecular mechanisms of prostate cancer progression will undoubtedly lead to the development of new molecular prognostic markers.

The nuclear factor-kappaB (NF-*κ*B) transcription factor family is composed of five structurally related members that possess an N-terminal Rel homology (RH) domain involved in protein–protein interactions and DNA binding (Ghosh and Karin, 2002). The NF-*κ*B proteins can be divided into two classes based on sequences C-terminal to their RH domain. The class 1 includes Rel (c-Rel), RelA (p65), and RelB proteins that are characterised by a C-terminal transactivation domain. NF-*κ*B1 (p50 and its precursor p105) and NF-*κ*B2 (p52 and its precursor p100) form the second class; p105 and p100 contain inhibitory C-terminal ankyrin repeats that are cleaved to create transcriptionally active p50 and p52 proteins. Rel/NF-*κ*B proteins exist as homo- and heterodimers. The best-studied dimers include the canonical (RelA/p50) and noncanonical (RelB/p52) NF-*κ*B complexes. Normally, the activation of NF-*κ*B requires signals that converge to I*κ*B kinases (IKKs). In the canonical pathway, the IKK complex (IKK*α*, *β*, *γ*) phosphorylates I*κ*Bs, which are then ubiquitinated and targeted for proteasome-dependent degradation. In the noncanonical pathway, IKK*α* regulates the processing of the p100 precursor. As a result of the activation of either pathway, NF-*κ*B dimers translocate to the nucleus and activate the expression of various genes involved in cell growth, differentiation, inflammatory responses, and the regulation of apoptosis (Aggarwal, 2004).

The NF-*κ*B pathways and genes are frequently altered in lymphoid and nonlymphoid cancers (Rayet and Gelinas, 1999). For instance, chromosomal alterations involving the c-Rel and *NF-κB2* genes have been detected in several B- and T-cell lymphomas (Liptay *et al*, 1997; Munzert *et al*, 2000). It has been shown that constitutively nuclear and active RelA/p50 dimers can prevent cell death by apoptosis in many cancer cell types after chemotherapy, radiotherapy, or TNF-*α* treatment (Barkett and Gilmore, 1999; Baldwin, 2001) and have also been detected in the nuclei of pancreatic and breast carcinomas (Sovak *et al*, 1997; Wang *et al*, 1999). More recently, constitutive activation of NF-*κ*B (RelA/p50) has been detected in androgen-independent prostate cancer cell lines as well as in prostate cancer tissues (Chen and Sawyers, 2002; Gasparian *et al*, 2002; Suh *et al*, 2002; Ayala *et al*, 2004; Ross *et al*, 2004; Shukla *et al*, 2004; Sweeney *et al*, 2004) and appears to promote cell growth, survival, and metastasis (Huang *et al*, 2001; Hodge *et al*, 2003; Levine *et al*, 2003; Ling *et al*, 2003). In particular, we and others have shown that nuclear localisation of RelA in primary prostate tumours is linked to poor clinical outcomes (Lessard *et al*, 2003; Ayala *et al*, 2004; Fradet *et al*, 2004; Ismail *et al*, 2004; Ross *et al*, 2004).

While RelA has become a candidate prognostic marker of prostate cancer progression, little is known about the expression and the subcellular localisation of other NF-*κ*B subunits. The present study provides the first large-scale immunohistochemical characterisation of all NF-*κ*B transcription factors in tissue-arrayed prostate cancer specimens. Our results show that all NF-*κ*B subunits are expressed in prostate tissues and that except for c-Rel, they are often detected in the nucleus of cancer cells. Interestingly, nuclear RelA and p50 are less frequently detected in the same cores than all other subunit combinations. In addition, the number of nuclear RelB-positive cores increased significantly with increasing Gleason scores. These results suggest that most NF-*κ*B transcription factors, especially noncanonical subunits, may play a role in the progression of prostate cancer.

## MATERIALS AND METHODS

### Prostate tissue microarray preparation

Archival formalin-fixed, paraffin-embedded (FFPE) human prostate tumour specimens were used to construct a human prostate tissue array of benign, high-grade prostatic intraepithelial neoplasia (PIN), and cancer (adenocarcinoma) tissue samples. The array was subdivided into normal, PIN, low Gleason (2, 2/3, 3), and high Gleason (3/4, 4, 4/5, 5) cores. Benign tissue cores were obtained from transition zone biopsies of radical prostatectomy specimens. Core tissue prostatectomy specimens (0.6 mm diameter) were obtained from preselected regions of individual paraffin-embedded donor blocks and precisely arrayed into a new recipient paraffin block with a tissue arrayer (Beecher Instrument, Silver Spring, MD, USA). After the block construction was completed, 5 *μ*m sections were cut with a microtome using an adhesive-coated tape sectioning system (Instrumedics, Hackensack, NJ, USA) to support the adhesion of the array elements.

### Immunohistochemical staining

Tissue sections were immunostained for NF-*κ*B transcription factors using the biotin–streptavidin immunoperoxidase method as previously described (Fradet *et al*, 2004). Briefly, FFPE tissue array slides were initially deparaffinised with toluene and rehydrated through graded ethanol. Endogenous peroxidase activity was blocked with 3% hydrogen peroxide for 10 min. An antigen retrieval technique was applied by boiling slides for 10 min at 95°C in a 0.01 M sodium citrate buffer, pH 6.0. The staining of c-Rel was enhanced using 1 mM EDTA (pH 8.0) instead of citrate. Tissue sections were incubated with a protein blocking serum-free reagent (Dako Diagnostics, Mississauga, ON, Canada) for 15 min to block nonspecific binding. The NF-*κ*B subunit expression was studied using mouse monoclonal NF-*κ*B RelA (p65) F-6, rabbit polyclonal RelB C-19, mouse monoclonal c-Rel B6, rabbit polyclonal NF-*κ*B p50 NLS (all from Santa Cruz Biotechnology, Santa Cruz, CA, USA), and mouse monoclonal NF-*κ*B p52 (Upstate Biotechnology, Lake Placid, NY, USA). Primary antibodies were applied at a concentration of 1 : 10 (c-Rel) or 1 : 50 (RelA, RelB, p50, p52) in PBS and were incubated for 2 h at room temperature. Immune complexes were revealed using a biotin-conjugated broad-spectrum secondary antibody (20 min), then streptavidin-peroxidase conjugate for 20 min (DakoCytomation, Mississauga, ON, Canada), followed by chromogen (0.06% 3,3-diaminobenzidine tetrahydrochloride, 0.01% hydrogen peroxide in PBS) for 5 min. Sections were counterstained with Mayer's haematoxylin, dehydrated, and then mounted. Negative controls were performed by omitting the primary antibody.

### Analysis

Damaged or nonrepresentative tissue cores (e.g., absence of epithelial tissue) were excluded from the analysis, leaving 344 (RelA), 346 (RelB), 369 (c-Rel), 343 (p52), and 344 (p50) cores from 75 patients. Cores were scored either as positive (1) or negative (0) for unequivocal brown nuclear NF-*κ*B staining in at least 5% of cancer cells. Clinical data were also available in a subset of samples in which preoperative serum prostate-specific antigen (pPSA), pathologic Gleason score (sum), and pathologic stage were available for 41, 47, and 46 patients, respectively. All statistical tests were performed using SPSS, version 10 (SPSS, Chicago, IL, USA).

## RESULTS

### Nuclear factor-kappaB subunit expression and subcellular localisation in prostatic tissues

**Figure 1:**
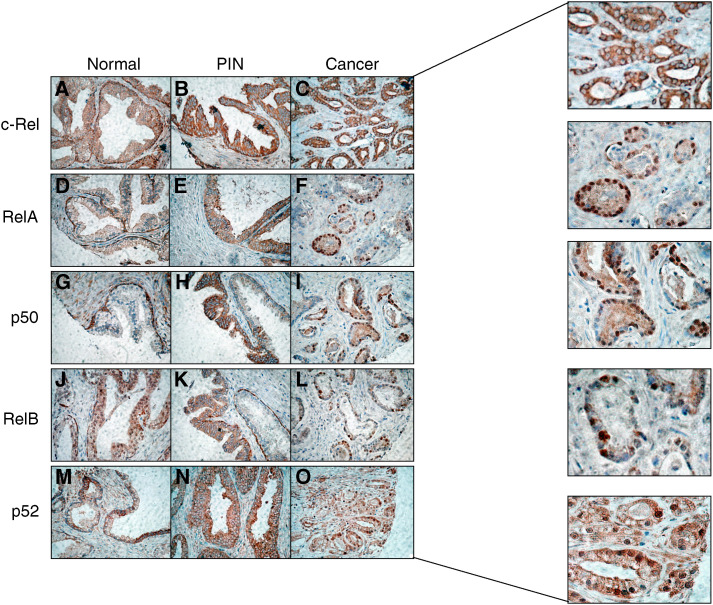
Immunohistochemical detection of c-Rel (**A**–**C**), RelA (**D**–**F**), p50 (**G**–**I**), RelB (**J**–**L**), and p52 (**M**–**O**) in normal, PIN, and cancerous prostate tissues (× 400). Note nuclear RelB staining in a subset of normal glands (**J**). Also note a mixture of weak and strong p52 cytoplasmic staining in normal glands (**M**). Enlargements are provided to more clearly distinguish the subcellular localisation in cancer specimens.

As shown in Figure 1, all NF-*κ*B subunits were expressed in prostatic tissues. Most benign glands were characterised by a strong cytoplasmic staining of the basal cell constituent and a variable cytoplasmic staining of the secretory (luminal) cells. Nuclear factor-*κ*B nuclear expression was usually not detected in benign tissues with the exception of a few cores in which RelB (two out of 40) and p52 (one out of 39) were localised in the nucleus of secretory cells. Prostatic intraepithelial neoplasia samples expressed weak to strong cytoplasmic levels of NF-*κ*B subunits and only one core (one out of 23) demonstrated RelA nuclear staining. Finally, all NF-*κ*B proteins were present in the cytoplasm of cancer cells and all except c-Rel were detected in the nuclei of cancer tissues at variable frequencies. Table 1 compares the frequencies of NF-*κ*B nuclear staining between normal, PIN, and cancer specimens. In all cases, the frequency of positive cores was significantly higher in cancer tissues as compared to noncancerous tissues. Within cancer specimens, nuclear RelB expression was the most commonly detected followed by RelA, p52, and p50. When cancer tissues were divided into low- to intermediate-grade (Gleason scores 2, 2/3, 3) and high-grade (Gleason scores 3/4, 4, 4/5, 5) tumours, no statistically significant difference was observed between the two groups, but there was a trend towards increased RelB nuclear staining in high-grade specimens (Table 2).

### Nuclear detection of several NF-*κ*B subunits in individual cores

**Figure 2:**
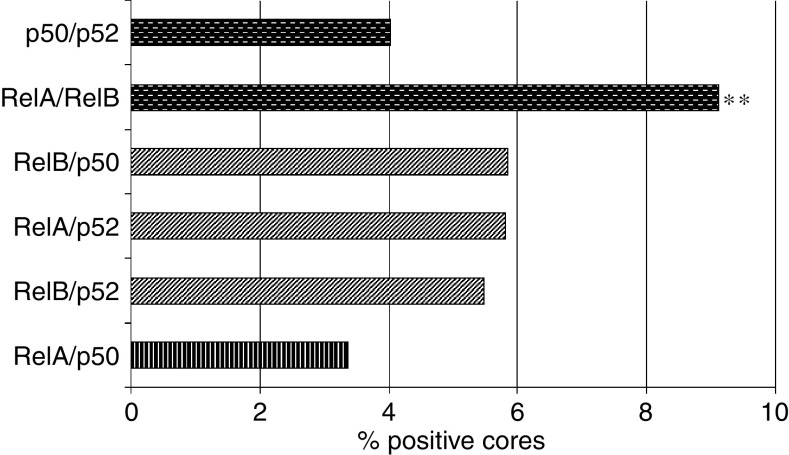
Frequencies of NF-*κ*B subunit combinations in the prostate cancer cores. The vertical pattern is for the canonical RelA–p50 pair. Noncanonical combinations are represented as follows: the diagonal pattern is for other class 1/class 2 dimers, and the dashed pattern is for class 1/class 1 or class 2/class 2 dimers. ^**^P<0.01 *χ*^2^ between RelA–p50 and RelA–RelB.

The NF-*κ*B proteins function as homo- and heterodimers. To assess the relative importance of these dimers in prostatic tissues, we calculated the proportion of cores in which both subunits of an NF-*κ*B heterodimer were detected in the nucleus of prostatic cells (Figure 2). Importantly, although all subunit combinations were observed in cancer cores, the canonical RelA–p50 pair was less frequently detected than other subunit pairs. In particular, the proportion of cores positive for both RelA and RelB (class 1 subunits) was significantly higher than that of cores positive for RelA and p50 (*P*=0.008).

### Relationship between nuclear NF-*κ*B and clinical parameters in prostate cancer patients

We performed statistical analyses to identify possible correlations and/or associations between NF-*κ*B nuclear staining and pPSA levels, pathological stage, and patient's Gleason score. No significant relationship was obtained between nuclear NF-*κ*B and pPSA or pathological stage. In contrast, the number of nuclear RelB-positive cores increased significantly with increasing Gleason scores (Spearman coefficient: 0.167; *P*=0.018; Table 3).

## DISCUSSION

Although prostate cancer can be detected in its early stages, it is still difficult to predict whether it will remain latent or progress to an advanced, metastatic disease. This is largely due to the lack of clinically proven molecular markers of prostate cancer progression. There are hundreds of candidate genes and proteins under investigation but few have been validated in multivariate analyses (Tricoli *et al*, 2004). We and others have recently shown that RelA, a member of the NF-*κ*B transcription factor family, is an independent predictor of biochemical recurrence (Ayala *et al*, 2004; Fradet *et al*, 2004; Ross *et al*, 2004). In addition, we have detected high levels of nuclear RelA staining in lymph node metastases and in patients who developed bone metastases (Lessard *et al*, 2003; Ismail *et al*, 2004). Hence, RelA appears to play a role in the progression of prostate cancer but little is known about the status of other NF-*κ*B family members, and no simultaneous analysis of the subcellular localisation of these subunits has been previously reported in prostate tissues.

In the present study, we characterised the expression and the subcellular localisation of RelA, p50, RelB, p52, and c-Rel. All subunits are expressed in prostatic tissues, and all but c-Rel can be detected in the nucleus of prostatic cells, more frequently in cancerous tissues. This suggests that both canonical (RelA, p50) and the noncanonical (e.g., RelB, p52) NF-*κ*B subunits can be activated in prostate cancer cells, whereas c-Rel remains inactive. Notably, a recent study reported a negative or nonspecific p50 expression in prostate tumours (Shukla *et al*, 2004). Conversely, we observed high levels of cytoplasmic p50 expression in cancer tissues. This discrepancy may be related to use of different antibodies.

The NF-*κ*B subunits can potentially form six heterodimers: RelA/p50, RelB/p52, RelA/p52, RelB/p50, RelA/RelB, and p50/p52. Importantly, each dimer may differently affect the cellular transcriptome, thus influencing disease progression. While in this study we did not directly assess the presence of specific heterodimers in prostate cancer specimens, our results provide an indication of those subunits present in the nucleus of cancer cells. Our results clearly demonstrate that within a given sample, we were more likely to find nuclear RelA and RelB, while the nuclear localisation of RelA and p50 was less frequently observed. If these subunits are relocalised within the same cell, this would suggest that noncanonical dimers are more frequently activated in prostate cancer, which is in contrast with the more usually observed activation of the canonical pathway in other cancers. Since RelA–RelB dimers are not regulated by I*κ*B proteins, they may act together or in concert with other transcription factors to attract histone acetyl transferases and cofactors and constitutively drive the transcription of specific genes. On the other hand, it has been reported that RelB forms transcriptionally inactive complexes with RelA in mouse embryonic fibroblasts (Marienfeld *et al*, 2003). In this model, RelB sequesters RelA and thereby represses RelA-dependent transcription. Whether RelA–RelB complexes exist in prostate cancer cells remains to be elucidated, but our results justify further investigation.

There is also a possibility that the RelB–p52 combination is more frequent than the canonical RelA–p50 pair (Figure 2). Given that RelB and p52 have been shown to be activated in other tumour types such as B- and T-cell lymphomas as well as in breast carcinoma (Dejardin *et al*, 1998; Rayet and Gelinas, 1999; Cogswell *et al*, 2000), they may also play a role in prostate cancer development and progression. Interestingly, both nuclear RelB and nuclear p52 were also detected in a subset of benign tissues (Table 1). However, it is important to note that all benign tissues collected were from cancerous prostates. Consequently, the nuclear localisation of RelB and p52 may evoke a normal transient activation, but may also reflect a cellular response to the tumour environment or a transitional state towards transformation. To test this hypothesis, we are presently characterising the activation status of NF-*κ*B subunits in cancer-free prostate specimens obtained from young male autopsies.

Correlation analyses between NF-*κ*B subunits and relevant clinical parameters revealed a significant relationship between RelB nuclear expression and patient's Gleason score (Table 3). Similarly, there was also a trend towards higher RelB nuclear staining in high-grade cancer tissue cores (Table 2). Whether RelB or other subunits confer a more aggressive phenotype to prostatic carcinoma remains to be determined. To this end, we are presently expanding the analysis to more clearly define the role of RelB in prostate cancer progression.

The absence of a correlation between RelA nuclear expression and patient's Gleason score confirms our previous observations (Lessard *et al*, 2003). Despite this, RelA acts as an independent predictor of patient's outcome (Fradet *et al*, 2004), which suggests that p50 and p52 should also be analysed to assess their potential as prognostic markers.

To our knowledge, this is the first large-scale immunohistochemical characterisation of NF-*κ*B transcription factors in the same cohort of prostate cancer patients. Our results not only provide further evidence for a role of the canonical NF-*κ*B pathway in prostate cancer, but also suggest a potential role for other NF-*κ*B subunits and pathways. Studies are ongoing to validate the usefulness of specific NF-*κ*B subunits, or combinations, as prognostic markers.

## Figures and Tables

**Table 1:** Nuclear localisation of NF-*κ*B transcription factors in prostatic tissue cores

	**RelA**			**p50**			**RelB**			**p52**		
**Tissue type**	***N***	**No. positive (%)**	***P***	***N***	**No. positive (%)**	***P***	***N***	**No. positive (%)**	***P***	***N***	**No. positive (%)**	***P***
Normal	39	0 (0)		37	0 (0)		40	2 (5.0)		39	1 (2.6)	
PIN	23	1 (4.3)		21	0 (0)		24	0 (0)		24	0 (0)	
Cancer	282	44 (15.6)	0.001	285	30 (10.5)	0.004	282	75 (26.6)	<0.001	281	30 (10.7)	0.025

*χ*^2^ was used to test the association of nuclear NF-*κ*B expression with tissue type (cancer *vs* noncancer).

*P*-value less than 0.05 was considered as significant.

NF-*κ*B=nuclear factor-*κ*B; PIN=prostatic intraepithelial neoplasia.

**Table 2:** Nuclear localisation of NF-*κ*B transcription factors in low to intermediate (2, 2/3, 3) and high (3/4, 4, 4/5, 5) Gleason grade scores

	**RelA**			**p50**			**RelB**			**p52**		
**Gleason group**	***N***	**No. positive (%)**	***P***	***N***	**No. positive (%)**	***P***	***N***	**No. positive (%)**	***P***	***N***	**No. positive (%)**	***P***
Low	170	24 (14.1)		174	16 (9.2)		169	40 (23.7)		167	16 (9.6)	
High	112	20 (17.9)	0.405	111	14 (12.6)	0.431	113	35 (31.0)	0.104	114	14 (12.3)	0.555

*χ*^2^ was used to test the association between nuclear NF-*κ*B and tissue grade. *P*-value less than 0.05 was considered as significant. NF-*κ*B=nuclear factor-*κ*B.

**Table 3:** Correlation between NF-*κ*B nuclear localisation and patient's Gleason scores

**Subunits**	**Coefficient**	***P*-value**
RelA	0.078	0.272
p50	−0.018	0.803
RelB	0.167	0.018*
p52	−0.019	0.793

Spearman's *σ* coefficient was used to test the relationship between nuclear NF-*κ*B expression and Gleason scores.

* *P*-value less than 0.05 was considered as significant. NF-*κ*B=nuclear factor-*κ*B.
